# Novel Strategies for Efficient Production and Delivery of Live Biotherapeutics and Biotechnological Uses of *Lactococcus lactis*: The Lactic Acid Bacterium Model

**DOI:** 10.3389/fbioe.2020.517166

**Published:** 2020-11-04

**Authors:** Laísa M. Tavares, Luís C. L. de Jesus, Tales F. da Silva, Fernanda A. L. Barroso, Viviane L. Batista, Nina D. Coelho-Rocha, Vasco Azevedo, Mariana M. Drumond, Pamela Mancha-Agresti

**Affiliations:** ^1^Laboratory of Cellular and Molecular Genetics, Federal University of Minas Gerais, Belo Horizonte, Brazil; ^2^Departamento de Ciências Biológicas, Centro Federal de Educação Tecnológica de Minas Gerais, Belo Horizonte, Brazil; ^3^FAMINAS - BH, Belo Horizonte, Brazil

**Keywords:** *Lactococcus lactis*, genetic engineering, biotherapeutic molecules, mucosal immunity, safe for consumption

## Abstract

Lactic acid bacteria (LAB) are traditionally used in fermentation and food preservation processes and are recognized as safe for consumption. Recently, they have attracted attention due to their health-promoting properties; many species are already widely used as probiotics for treatment or prevention of various medical conditions, including inflammatory bowel diseases, infections, and autoimmune disorders. Some LAB, especially *Lactococcus lactis*, have been engineered as live vehicles for delivery of DNA vaccines and for production of therapeutic biomolecules. Here, we summarize work on engineering of LAB, with emphasis on the model LAB, *L. lactis*. We review the various expression systems for the production of heterologous proteins in *Lactococcus* spp. and its use as a live delivery system of DNA vaccines and for expression of biotherapeutics using the eukaryotic cell machinery. We have included examples of molecules produced by these expression platforms and their application in clinical disorders. We also present the CRISPR-Cas approach as a novel methodology for the development and optimization of food-grade expression of useful substances, and detail methods to improve DNA delivery by LAB to the gastrointestinal tract. Finally, we discuss perspectives for the development of medical applications of recombinant LABs involving animal model studies and human clinical trials, and we touch on the main safety issues that need to be taken into account so that bioengineered versions of these generally recognized as safe organisms will be considered acceptable for medical use.

## Introduction

Lactic acid bacteria (LAB) constitute a heterologous group of non-sporulating, Gram-positive, microaerophilic, non-mobile, and catalase-negative microorganisms (Makarova and Koonin, 2007). The main characteristic of this group is the ability to produce lactic acid as a product of carbohydrate fermentation (Wang et al., 2016; Mokoena, 2017). These bacteria are classified as homofermentative or heterofermentative based on the final product of their fermentation. Lactic acid is the main product of glucose fermentation in the homofermentative group, while the heterofermentative group, in addition to lactic acid, produces other substances, such as carbon dioxide, acetic acid and ethanol (Carr et al., 2002). LAB organisms include various species of the genera *Lactococcus*, *Lactobacillus*, *Oenococcus*, *Enterococcus*, *Streptococcus*, *Pediococcus*, *Tetragenococcus*, *Vagococcus*, *Leuconostoc*, *Carnobacterium*, *Sporolactobacillus*, and *Weissella* (Stiles and Holzapfel, 1997).

On account of their long-term safe use in human nutrition, the majority of these bacteria have been recognized as safe, receiving the GRAS (Generally Recognized As Safe) status by the United States Food and Drug Administration (FDA), being commonly used for fermentation in the food industry, and as probiotic food supplements due to their beneficial health effects (Mao et al., 2016).

Probiotics are defined as “*live microorganisms that, when administered in adequate amounts, confer a health benefit on the host*” (Hill et al., 2014), playing an important role in overall health and for preventing infections (Reid et al., 2003; Klaenhammer et al., 2005). Some of the beneficial effects of probiotic bacteria include: (i) they restore the intestinal microbiota (Shi et al., 2017); (ii) they can eliminate pathogens (Halder et al., 2017), they induce production of β-defensins by host Paneth cells (Karacaer et al., 2017), they have antimicrobial activity, such as by organic acid production, modifying the pH (Karacaer et al., 2017; Kimelman and Shemesh, 2019), they produce bioactive metabolites, such as hydrogen peroxide (Karacaer et al., 2017), they produce bacteriocins and microcins (Karacaer et al., 2017; Gaspar et al., 2018); (iii) they compete with pathogens for nutrients (Deriu et al., 2013); (iv) they compete for host-cell adhesion receptors (Walsham et al., 2016); (v) they can reduce the activity of pathogen-produced toxins (Ripert et al., 2016); (vi) they produce molecules capable of interfering with *Quorum Sensing* and biofilm production (Shokri et al., 2018; Wasfi et al., 2018), contributing to the elimination of bacteria that penetrate into the mucus layer (Hooper and Macpherson, 2010); and (vii) they induce IgA production by the host (Hemarajata and Versalovic, 2013).

Additionally, probiotic bacteria can enhance the intestinal barrier against foreign antigens by maintaining the integrity of epithelial cell tight-junctions (Blackwood et al., 2017), and by inducing mucin production, which provides protection against antigens and foreign molecules, and also acts as a lubricant for intestinal motility. As mucus is the first barrier that intestinal bacteria encounter, pathogens need to penetrate it during an infection to reach the epithelial cells (Phillipson et al., 2008; Aliakbarpour et al., 2012). In addition, LAB may also be able to inhibit activation of the NF-κβ pathway (Kaci et al., 2011; Gao et al., 2015), presenting immunomodulatory properties such as stimulation of both innate and adaptive host immunity (Hemarajata and Versalovic, 2013; Underwood, 2016). These bacteria can also produce and secrete metabolites with anti-inflammatory properties capable of preventing or relieving symptoms of gastrointestinal problems, such as inflammatory bowel diseases (Huebner and Surawicz, 2006; Plaza-Díaz et al., 2017; De Jesus et al., 2019) and autoimmune diseases (de Oliveira et al., 2017).

In order to potentialize the beneficial effects of probiotic strains, research based on genetic engineering techniques has been conducted on LAB aiming at a broad spectrum of activities, providing candidate strains that could be used in various industrial sectors, including food and medicine production. LAB have been bioengineered for the production of diverse molecules of biotechnological interest, such as prophylactic compounds, pro-inflammatory molecules, and other useful substances, including bioactive peptides, cytokines, enzymes, and allergens (Wells and Mercenier, 2008; Wells J., 2011). Some new applications of LAB, include mucosal vaccines (Steidler et al., 2003; Pereira et al., 2015; Mancha-Agresti et al., 2017) and production of heterologous proteins (Saraiva et al., 2015; Carvalho R. D. D. O. et al., 2017). Nonetheless, the long-term effects of these products on host health have not been evaluated.

Among the LAB, *Lactococcus lactis*, is the most well characterized species and figures as the model organism for engineering studies of this group due to its economic importance in cheese production, and due to the ease with which it can be grown and manipulated. One important characteristic of this strain is its inability to colonize the human gastrointestinal tract (GIT); however, it can resist gastric acid and bile juice, allowing survival and transit through the GIT (Wells and Mercenier, 2008; Wells J. M., 2011; Song A. A.-L. et al., 2017). In addition, *L. lactis* was the first species of LAB to have its genome fully sequenced, which has allowed a better understanding of its genetic and physiological mechanisms (Poquet et al., 1998; Duwat et al., 2000; Ravn et al., 2000; Bolotin et al., 2001; Nouaille et al., 2003; Mills et al., 2006).

Bioengineered *L. lactis* strains have mainly been used as producers of heterologous proteins and as a vehicle for delivery of DNA vaccines.

## *Lactococcus lactis:* Heterologous Protein Production

Many expression systems have been developed for the production of recombinant proteins; among the prokaryotic systems, the highest protein production levels are obtained using *Escherichia coli* (Jana and Deb, 2005). However, the most commonly used strategy in this microorganism is intracellular protein production (periplasm or cytoplasm), which requires expensive and often problematic purification processes. In addition, endotoxins, such as lipopolysaccharides (LPS), must still be taken removed before the desired proteins can be safely administered to mammals (Morello et al., 2008). To avoid this problem with bacterial toxins, *L. lactis* stands out as an alternative microorganism for the production of molecules of biotechnological interest. Numerous proteins of viral, bacterial, and eukaryotic origin have already been produced using *L. lactis* (Nouaille et al., 2003; Le Loir et al., 2005).

Expression of heterologous proteins in *L. lactis* became feasible as a consequence of studies of its genetics and the development of more efficient molecular biology techniques (Nouaille et al., 2003). In order to obtain high and controlled levels of production, several vectors containing constitutive or inducible promoters were developed, forming, nowadays, the basis of all expression systems using *L. lactis.*

Some useful properties that make *L. lactis* an ideal candidate for the production of exogenous molecules are non-production of LPS and the absence of any other toxic metabolic product (Bolotin et al., 2001; Bahey-El-Din and Gahan, 2010). Another important feature of this species is the low number of secreted proteins; only one protein (USP45 – Unknown Secreted Protein of 45 kDa) is secreted in sufficient quantities to be detected in gel electrophoresis (van Asseldonk et al., 1990). This feature is important because it facilitates both the analysis and the purification of the proteins of interest. Moreover, some strains do not produce *PrtP* (subtilisin-like serine protease), an extracellular protease (Gasson, 1983), and two strains of this group (IL1403 and MG1363) are devoid of wild plasmids (Chopin et al., 1984).

Several heterologous protein expression and cell addressing systems have already been developed, not only for use in *L. lactis* but also for other LAB, such as *Lactobacillus* spp. and *Bifidobacterium* spp. (Norton et al., 1995; Piard et al., 1997; Dieye et al., 2001; Le Loir et al., 2001). Vectors carrying constitutive or inducible promoters were developed and used as key tools to increase production of heterologous proteins and control their production (Pontes et al., 2011). Among other factors, the level of expression of the recombinant gene is influenced by promoter properties; when comparing inducible and constitutive promoters, the former provide better control over recombinant protein expression (Plavec and Berlec, 2019).

The most commonly used expression systems in *L. lactis*, as well as in other LAB, includes P170 (Madsen et al., 1999), P_(Zn)zitR_ (Llull and Poquet, 2004), XIES (Xylose-Inducible Expression System) (Miyoshi et al., 2004), NICE (Nisin Controlled Expression System) (Mierau and Kleerebezem, 2005), SICE (Stress-Inducible Controlled Expression System) (Benbouziane et al., 2013), Zirex (Zinc-Regulated Expression System) (Mu et al., 2013), and ACE (Agmatine Controlled Expression System) (Linares et al., 2015a).

### P170 Expression System

**FIGURE 1:**
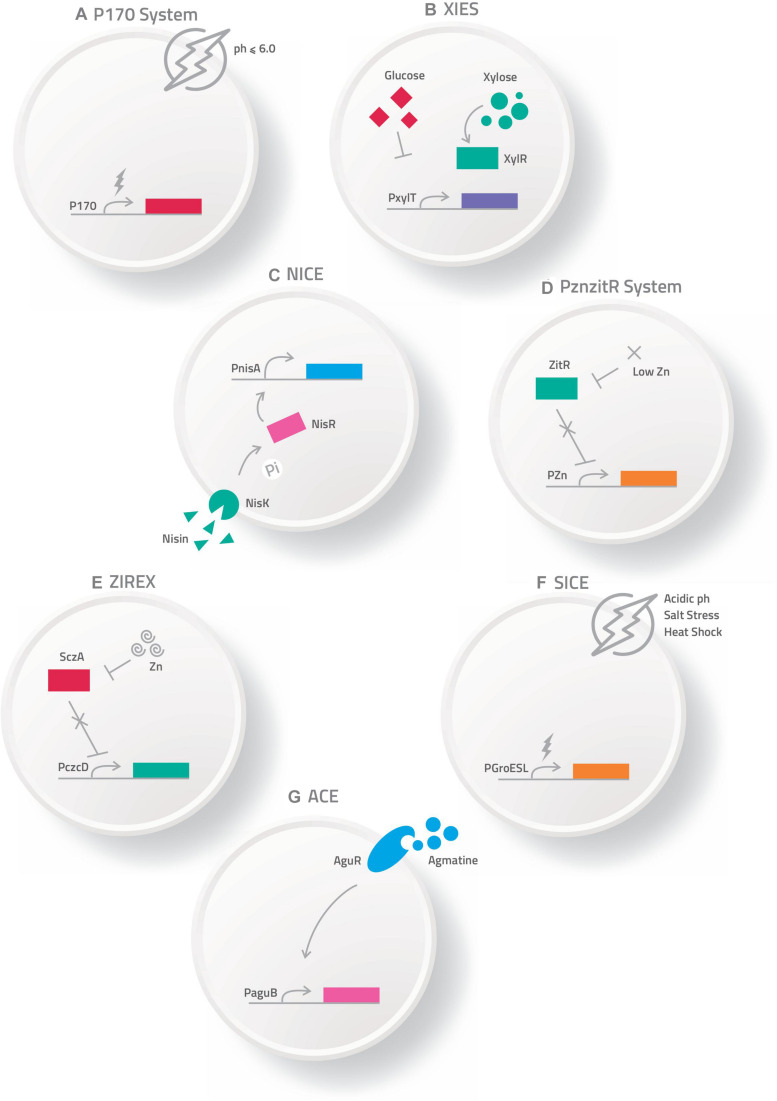
Schematic representation of some expression systems developed for the production of recombinant proteins. **(A)** P170 Expression system; **(B)** XIES – Xylose Inducible Expression System; **(C)** NICE – Nisin Controlled Expression System; **(D)** PZnzitR – Driven Heterologous Expression; **(E)** Zirex – Zinc-Regulated Expression System; **(F)** SICE – Stress Inducible Controlled Expression System; **(G)** ACE – Agmatine Controlled Expression System.

The P170 expression system is based on the P170 promoter from *L. lactis* MG1363 (Israelsen and Hansen, 1993), which is induced by the production of lactic acid in the stationary phase at pH 6.0–6.5, being considered an auto-induced promoter in the fermentation process (Figure 1A). This system has been used for production of more than 100 recombinant proteins (Madsen et al., 1999; Jørgensen et al., 2014).

This expression system was applied to the production of a chimeric protein, GMZ2, a combination of *Plasmodium falciparum* glutamate-rich protein (GLURP) and the merozoite surface protein 3 (MSP3), which can destabilize the malaria parasite life cycle. This hybrid protein has proven to be a promising strategy for malaria vaccine development due to its effectiveness and safety characteristics (Theisen et al., 2004; Esen et al., 2009). Currently, the GMZ2 malaria vaccine has concluded phase 2 of clinical trials, having been tested in African children and has proven to be well tolerated and effective in the target population, although some improvements in immunogenic formulations will be needed to allow widespread use (Sirima et al., 2016; Theisen et al., 2017).

Another study that concluded phase 3 of clinical trials in 2015 incorporated the CFP-10 antigen together with the ESAT-6 antigen (C-Tb), produced by *L. lactis*, in the development of a skin test for diagnosis of *Mycobacterium tuberculosis* infection that is not affected by BCG vaccination. The C-Tb protein showed high specificity (about 99.3%), providing a promising alternative for the skin test (Aggerbeck et al., 2013; Hoff et al., 2016; Ruhwald et al., 2017).

P170 was also used for heterologous expression of β-galactosidases in both *L. lactis* and *Bifidobacterium bifidum* for nutritional and therapeutic applications, since this enzyme is used by the food industry to produce galacto-oligosaccharides (GOS), prebiotic substances that have functional properties in food (Jørgensen et al., 2001; Schwab et al., 2010). Also, for the food industry, P170 and an optimized signal peptide (SP310mut2) have been used for the production and secretion of sucrose isomerase from *Enterobacter* sp., by *L. lactis*, to convert sucrose into isomaltulose, a functional sucrose substitute that is healthier than sucrose itself, being a low-glycemic and low-insulinemic molecule. The sucrose isomerase secreted by *L. lactis* MG1363 was able to convert sucrose to isomaltulose at a rate of up to 72% (Park et al., 2010).

### XIES – Xylose-Inducible Expression System

Miyoshi et al. (2004) developed an inducible system of heterologous expression and protein targeting based on the xylose permease gene promoter (PxylT) from *L. lactis* NCDO2118. This expression system, named XIES (Xylose-Inducible Expression System), can be easily switched on by the addition of xylose in the bacterial growth medium, activating the promoter; alternatively, PxylT is transcriptionally repressed in the presence of glucose (Miyoshi et al., 2004) (Figure 1B). This system has two different versions; the cytoplasmic version, in which the protein that is produced is maintained inside the bacterial cell and the secreted one, in which the protein is secreted and can be harvested from the supernatant.

The XIES system was successfully tested using the *Staphylococcus aureus* nuclease (*nuc*) sequence as a reporter gene; high cytoplasmic levels of this protein were obtained. When the *nuc* coding sequence was fused to the signal peptide (SP) of the lactococcal secreted protein Usp45 present in pXIES-SEC (secretory version of the XIES system), high levels of secreted protein were also obtained (Miyoshi et al., 2004).

The XIES system has been widely studied in many disease models. The *L. lactis* strain NCDO2118 harboring the XIES system (cytoplasmic and secreted versions) encoding the full-length IL-10 of *Rattus norvegicus* was used to evaluate its immunomodulatory effect. The cytoplasmic version used in a mouse model of ovalbumin (OVA) induced acute airway inflammation (Marinho et al., 2010) and modulated airway inflammation independent of the regulatory T cells (Treg). It also reduced eosinophil peroxidase activity (EPO), and IgE anti-OVA, interleukin 4 (IL−4) and chemokine (C-C motif) ligand 3 (CCL3) levels (Marinho et al., 2010).

Fermented milk preparation with *L. lactis* strain NCDO2118 (pXIES:SEC:*hsp65*) was orally administered in a murine model of Crohn’s Disease induced by trinitrobenzenesulfonic acid (TNBS) (del Carmen et al., 2011). Treated animals presented lower damage scores in histological analysis of the large intestine, along with decreased IFN-γ levels, little microbial translocation to the liver, and higher IL-10/IFN-γ, IL-10/IL-12, and IL-10/IL-17 ratios (del Carmen et al., 2011). Although the strain producing IL-10 in the cytoplasm showed a greater immunomodulatory potential in the murine lung inflammation model (Marinho et al., 2010), the IL-10 secreting strain conversely revealed more pronounced anti-inflammatory effects.

*Lactococcus lactis* strain secreting *Mycobacterium leprae* heat-shock protein HSP65 (pSEC:*hsp65)* (de Azevedo et al., 2012) was orally administrated to mice as treatment for auto-immune encephalomyelitis (EAE). This reduced inflammatory cell infiltrates and there were no signs of injury to the spinal cord, along with reduced IL-17 and increased IL-10 cytokine production in mesenteric lymph nodes and spleen cell cultures (Rezende et al., 2013). This recombinant strain was also used in experimental DSS (dextran sodium sulfate) induced colitis mice models (Gomes-Santos et al., 2017). Oral pretreatment prevented disease development by reducing pro-inflammatory cytokines (IFN-γ, IL-6, and TNF-α) and increased IL-10 production in colonic tissue (Gomes-Santos et al., 2017).

Another recombinant lineage of *L. lactis* NCDO2118 harboring the XIES system in its cytoplasmic version was constructed to produce human 15-lipoxygenase-1 (15-LOX-1), named *L. lactis* (pXIES:CYT:15-LOX-1) (Saraiva et al., 2015). This molecule participates in the oxidative metabolism of polyunsaturated fatty acids (PUFAs) (Brash et al., 1997), thus providing anti-inflammatory effects. Milk fermented by *L. lactis* (p XIES:CYT:15-LOX-1) was used to treat animals with IBD induced by TNBS. Carvalho R. D. et al. (2017) grew this same strain in their own culture medium and also tested it in a IBD mouse model, induced by DSS. Thus *L. lactis* (pXIES:CYT:15-LOX-1) is promising for treating intestinal epithelium affected by IBD disease (Saraiva et al., 2015; Carvalho R. D. et al., 2017).

### NICE – Nisin Controlled Expression System

This system is based on the expression of three genes (*nis*A, *nis*F, and *nis*R) that are involved in the production and regulation of the antimicrobial peptide nisin, naturally secreted by various strains of *L. lactis*. In this system, the membrane-located histidine kinase (*Nis*K) reacts to the signal inducer nisin and auto-phosphorylates, and then transfers a phosphorous group to the intracellular response regulator protein *Nis*R, which acts as a transcription activator of *nis*A/*nis*F and induces gene expression regulated by the p*Nis* promoter. Depending on the chemical signals, this protein can be expressed in the cytoplasm, on the cell membrane, or secreted into the culture medium (de Ruyter et al., 1996; Blatny et al., 2003; Mierau and Kleerebezem, 2005) (Figure 1C).

The NICE system includes three essential elements: (i) the hosts: Gram-positive bacteria that can express *nis*K and *nis*R (i.e., *Lactobacillus*, *Streptococcus*, *Enterococcus*, *L. lactis*); (ii) the plasmid vector: which includes a *nis*A or *nis*F promoter region allowing nisin induction; and (iii) the inductor nisin, which must be present during the log-growth phase at a concentration of 0.01 to 10 ng/mL. However, the NICE system can only be used with bacterial strains containing the genes *nis*R and *nis*K that encode the NisRK system, which is responsible for controlling the expression of the *nis*A gene via signal transduction (Zhou et al., 2006; Mancha-Agresti et al., 2015).

*Brucella abortus* is a worldwide zoonosis, and it is the main cause of abortion and infertility in cattle, also causing undulant fever in humans (Ribeiro et al., 2002). Vaccination against brucellosis for cattle and other ungulates depends on live attenuated strains; however, vaccination provokes abortion when administered to pregnant cattle. Recombinant *L. lactis* strains producing the *B. abortus* L7/L12 antigen were constructed using the NICE system. These recombinant *L. lactis* strains provoked high L7/L12 levels, demonstrating that recombinant *L. lactis* (pNICE:SEC:L7/L12) could be used as delivery vehicles to elicit both mucosal and systemic immune responses against brucellosis, providing a viable alternative to vaccination with live bacteria (Ribeiro et al., 2002).

In order to evaluate the mucosal and systemic antibody response to L7/L12, 1 × 10^9^ CFU of *L. lactis* (pNICE:CYT:L7/L12) recombinant strain were orally administered to BALB/c mice on days 0, 1, 2, 14, 15, 16, 28, 29, and 30. After the immunization protocol, on day 45, mice were challenged by intraperitoneal injection with 1 × 10^5^ CFU of *B. abortus* strain 2308. The live oral vaccine induced a strong mucosal response, evidenced by an antigen-specific response observed in feces of mice intragastrically immunized, providing moderate protection against challenge with strain 2308 of *B. abortus* (Pontes et al., 2003).

The azurin protein of the bacterium *Pseudomonas aeruginosa* is composed of 128 amino acids; this bacteriocin has anti-cancer properties. The azurin sequence was cloned into the pNZ8149 vector and transformed into *L. lactis* NZ3900, using induction with 1, 5, 10, 20, and 50 ng/mL of nisin for 3 h, Subsequently, after centrifuging, the pellet and the supernatant (SN) were separated. The SN was lyophilized and tested *in vitro* at concentrations of 2.5, 5.0 and 10 mg/mL. This SN had antimicrobial activity against *Escherichia coli* BC1402 and *Bacillus cereus* ATCC33019. It also had greater antiproliferative activity when compared to the SN of *Pseudomonas aeruginosa* that did not produce azurin. Yildiz and Askin (2019) concluded that this recombinant strain could be used as food biopreservative against *E. coli* and *B. cereus*, consequently constituting a potential therapeutic probiotic.

The NICE System was used to produce oncogenic viral protein E6 of HPV-16. This protein has been identified in infected cells and can serve as a tumor specific antigen. Potentially these tumor proteins could be used as immunotherapeutic alternatives for the treatment of cervical cancer associated with HPV (Taghinezhad-S et al., 2019). E6 HPV-16, isolated from an Iranian population and optimized, and the native E6 sequence, were cloned into the pNZ8123 vector (PnisA promoter, nisin-induced) and transformed into *L. Lactis* NZ9000. Western blotting confirmed protein expression. *In vivo* experiments were carried out with oral immunization of C57BL/6 mice, evaluating the potential of both recombinant strains for the treatment of tumors and to investigate the immune response. Both recombinant strains stimulated humoral and cellular immune responses. However, some parameters, such as inhibitory effects on tumor progress and survival of the animals, were superior when the optimized oncogene sequence was used. Thus, recombinant bacteria have potential for the development of an effective vaccine to treat HPV-16 patients (Taghinezhad-S et al., 2019).

Recently, a recombinant strain *L. lactis* NZ3900 was constructed to express the recombinant protein VP1, to immunize ducks against DHAV-3 virus, which causes viral hepatitis in these waterfowl, with a mortality rate of almost 100% in ducklings less than 3 weeks old. The ducks were orally immunized with recombinant *L. lactis* strain (5 × 10^11^ CFU/mL) and randomly sacrificed at 4, 6, and 8 days after the immunization protocol. This vaccination protocol was able to induce specific IgG and sIgA antibody production, as well as increased levels of IL-2, IFN-γ, IL-10, and IL-4. Thus, this recombinant *L. lactis* strain gave effective protection against DHAV-3 in ducklings, which could become an efficient strategy for the development of an oral vaccine for ducks (Song et al., 2019).

### ZINC – Inducible Expression Systems

#### P*_*Zn*_*zitR – Driven Heterologous Expression

Another expression system for heterologous protein production is P*_*Zn*_*zitR-driven heterologous expression. This system is based on the PZn promoter and the ZitR repressor from the *L. lactis* zit operon (zitRSQP), which is involved in zinc regulation. When zinc is abundant in the medium, the ZitR repressor binds to the PZn promoter and suppresses gene transcription. Alternatively, when zinc levels are low in the medium, the ZitR repressor becomes inactive and is unable to bind to the promoter, allowing RNA polymerase to bind to the PZn promoter and initiate transcription. Zinc depletion of medium can be achieved by addition of an EDTA chelator agent or by gradual zinc reduction in the culture medium due to bacterial growth (Llull and Poquet, 2004) (Figure 1D).

The functionality of the P*_*Zn*_*zitR-driven heterologous expression system was evaluated in *L. lactis* strain MG1363; the evaluation was based on production of two different reporter genes, *uspnuc* and *lacLM*, which encode a secreted nuclease protein derived from *S. aureus* and cytoplasmic β-galactosidase from *Leuconostoc mesenteroides*, respectively. The recombinant *L. lactis* carrying the P_*Zn*_zitR vector produces reporter proteins in Zn^2+^-containing medium after chelation with EDTA or by Zn^2+^ consumption during bacterial growth. In contrast, the expression of these proteins was strongly repressed in the presence of excess Zn^2+^ and absence of EDTA (Llull and Poquet, 2004).

#### Zirex – Zinc-Regulated Expression System

Zirex, is another zinc-inducible expression system developed in *L. lactis*. This system is based on the regulator protein *Scz*A and the promoter P_czcD_ of *Streptococcus pneumoniae* D39. In the absence of zinc, *Scz*A represses transcription; but when zinc is added to the medium the repressor moves along the nucleic acid sequences of the zinc-induced promoter P_czcD_, unblocking transcription (Figure 1E). This expression system was tested in *L. lactis* NZ9000 transformed with p*CZG* (a plasmid under Zirex control, containing SczA-P_czcD_) and cloned in the *GFP* ORF. Higher expression levels of GFP were observed (Mu et al., 2013). In the same study, to test simultaneous expression of different proteins regulated by different promoters, the pCZGM vector was constructed combining nisin and zinc expression systems, simultaneously, in which the P_nisA_ promoter (nisin) controlled the expression of mCherry and P_czcD_ (zinc) controlled the expression of GFP. After 2.5 h of induction with zinc and nisin, expression of GFP and mCherry was observed, although the simultaneous expression of both proteins with the pCZGM vector slightly reduced both fluorescent signals. This combination could be applied as a tool for overexpression of various types of proteins. Despite these potential advantages, no other studies involving this vector have been published (Mu et al., 2013).

### SICE – Stress-Inducible Controlled Expression System

The Stress-Inducible Controlled Expression System (SICE) is based on the groESL operon. This operon, described in *L. lactis* by Desmond et al. (2004), is able to positively induce protein synthesis of the cloned ORF after heat shock. Thus, after oral administration, the recombinant bacteria carrying the SICE vector reaches the GIT, where they face various types of stress, such as heat-shock, exposure to bile salts, and low pH; all these stresses are able to induce expression in this system (Figure 1F). The SICE system can also be used for intranasal applications, since the main stressing situation faced by bacteria in this route will be heat-shock. Thus, the most advantageous characteristic of this system is that it does not depend on external induction before administration since the adverse physiological conditions of the delivery site itself can induce expression (Benbouziane et al., 2013).

To validate the functionality of this vector, two recombinant strains of *L. lactis* were constructed: *L. lactis* delivering IL-10 and *L. lactis* delivering HPV-16 E7 (human papillomavirus type-16) antigen. Two routes of administration were also evaluated: the oral route for therapy in the Dinitrobenzene Sulfonic Acid (DNBS)-induced colitis model by administration of recombinant *L. lactis* SICE:*IL-10* and the intranasal route applied to a model of vaccination against HPV-16-induced tumors. The resulting IL-10 secretion reduced various colitis parameters, with potential for clinical trials to treat colitis. In mice vaccinated with recombinant *L. lactis* HPV-16 the tumor size was reduced (∼1 cm^3^); furthermore, there was no mortality in the treatment group, while mortality was about 16% in the control group (Benbouziane et al., 2013).

Another study also evaluated the protective effects of *L. lactis* delivering IL-10 in a TNBS-induced chronic colitis model using two different expression systems: SICE, based on heterologous expression of IL-10, and pValac vectors (see next section), a DNA vaccine vector harboring the IL-10 cDNA cassette. Both delivery systems restored the intestinal IL-10 levels of treated mice, inoculated with TNBS, having similar anti-inflammatory effects, preventing weight loss and reducing damage scores in the large intestine when compared with untreated mice (del Carmen et al., 2014).

Martín et al. (2014) demonstrated the beneficial effects of the SICE system, which is based on secretion of IL-10 in a low-grade inflammation model characterized by two episodes of DNBS-challenge in mice. The main beneficial results consisted of a significant decrease in intestinal hyperpermeability, a decrease in pro-inflammatory cytokines (IL-13, IL-1α, and IL-6) and in inflammatory status, with restoration of adherent junction (AJ) and tight junction (TJ) proteins in inflamed mice treated with the recombinant strain in comparison with untreated mice (Martín et al., 2014).

### ACE – Agmatine Controlled Expression System

This system is based on expression of the agmatine deiminase (AGDI) operon (*agu*R, *agu*B, *agu*D, *agu*A, and *agu*C) of *L. lactis* subsp. *cremoris* CECT 8666, which encodes the enzymatic activities involved in the catabolism of agmatine to putrescine (putrescine biosynthesis pathway). In this system, the regulatory transmembrane protein *agu*R, in response to extracellular agmatine supplementation, acts as a transcriptional activator system of the *agu*B promoter, which induces protein overproduction of the recombinant target gene (Linares et al., 2013, 2015a,b) (Figure 1G).

The functionality of the ACE system was evaluated in *L. lactis* NZ9000 by cloning the *gfp* ORF (pACE:*GFP*), which encodes the GFP reporter protein, and the *pep* ORF (pACE:pep) of *Myxococcus xanthus*, which encodes prolyl-endopeptidase, an enzyme of biomedical interest that is able to degrade immunotoxic peptides produced by the gastrointestinal breakdown of gluten. Production of these proteins was evaluated using a range of agmatine concentrations (from 0 to 60 mM). A significant increase in GFP protein fluorescence was observed after induction with agmatine at low concentrations (10^–5^ mM), with a maximum induction level at 0.5 mM agmatine. Also, prolyl-endopeptidase production and activity were observed even with the lowest agmatine concentrations tested (0.001 mM to 0.1 mM). Thus, the ACE expression system is highly inducible and is a potential candidate for large-scale production of a recombinant target protein in *L. lactis* (Linares et al., 2015a).

All the systems for production of recombinant proteins mentioned above involve cloning the suitable open reading frame of the gene of interest into an expression vector under the control of an inducible promoter. To obtain efficient expression of the cloned sequence, several aspects are involved, including correct protein folding, ideal expression of signals at transcription and translation levels, cell growth characteristics, protein toxicity, and codon usage (Schumann and Ferreira, 2004).

Though more than one codon is able to encode the same amino acid, natural selection acting on translational accuracy and efficiency of protein expression can result in non-random usage of specific codons (Akashi, 1994; Stoletzki and Eyre-Walker, 2006). As is generally understood, codon use bias among different species can differ, even among different genes from a single genome (Batard et al., 2000; Wei et al., 2014), reflecting the different GC contents of these organisms (Escarnís and Salas, 1982; Takkinen et al., 1983; Winter et al., 1983). It can be a barrier for efficient expression and should be considered when engineering Gram-negative bacterial proteins into Gram-positive species. To overcome this impediment, one can optimize codon usage bias by replacing some codons with those more frequently used by the host organisms. Currently, gene synthesis companies optimize the gene sequence before synthesis, using specific software to help reduce translation problems.

Inducible promoters allow greater control of protein expression by the user, and are therefore preferred over constitutive promoters. Nevertheless, for production on an industrial scale, the inclusion of an inducer can make the industrial process more expensive and require more time to produce the molecules of interest (Morello et al., 2008). In addition, many expression systems still have antibiotic resistance genes as selection markers, making them unsafe for human consumption and use in the pharmaceutical industry (Plavec and Berlec, 2020).

In summary, many vectors for heterologous protein production have been developed to be expressed by *L. lactis*. Most of them have been tested on various diseases, especially diseases affecting the GIT, often with good results. These systems have applications in the pharmaceutical, and food industries and also for medical use. They provide effective alternatives to treat various types of inflammatory diseases, demonstrating that the techniques for developing recombinant strains have therapeutic relevance (Table 1).

**TABLE 1:** Examples of engineered *Lactococcus lactis* as a delivery vector for therapeutic purposes.

Plasmid-encoded systems	Disease targeted or pathogen	Antigen or protein	Experimental model	References
p170	Malaria	GMZ2	Human	Sirima et al. (2016); Theisen et al. (2017)
	*Mycobacterium tuberculosis*	CFP-10 + ESAT-6	Human	Aggerbeck et al. (2013); Hoff et al. (2016); Ruhwald et al. (2017)
	Nutritional or therapeutic applications	B -galactosidases	–	Schwab et al. (2010)
XIES	Airway inflammation	IL-10	BALB/c mice	Marinho et al. (2010)
	Crohn’s disease	IL-10	BALB/c mice	del Carmen et al. (2011)
	Auto-immune encephalomyelitis (EAE)	Hsp65	C57BL/6 mice	Rezende et al. (2013)
	Colitis	Hsp65	C57BL/6 mice	Gomes-Santos et al. (2017)
	IBD	15-lipoxygenase-1 (15-LOX-1)	BALB/c mice	Saraiva et al. (2015)
NICE	*Brucella abortus*	L7/L12	BALB/c mice	Pontes et al. (2003)
	Viral hepatitis	VP1	BALB/c mice Ducks	Song et al. (2019)
	HPV-16	E6 opitE6	C57BL/6 mice	Taghinezhad-S et al. (2019)
SICE	Colitis	IL-10	C57BL/6 mice	Benbouziane et al. (2013)
	HPV-16 induced tumors	HPV-16 E7	C57BL/6 mice	Benbouziane et al. (2013)
	Chronic colitis	IL-10	BALB/c mice	del Carmen et al. (2014)
	IBD	IL-10	C57BL/6 mice	Martín et al. (2014)
pValac	IBD	IL-10	BALB/c mice	del Carmen et al. (2014); Zurita-Turk et al. (2014)
	Crohn’s disease	IL-4	BALB/c mice	Souza et al. (2016)
	Colitis	Anti-TNFα scFv	BALB/c mice	Chiabai et al. (2019)
	*Mycobacterium tuberculosis*	ESAT-6	BALB/c mice	Pereira et al. (2015)
	*Mycobacterium tuberculosis*	Ag85A	C57BL/6 mice	Mancha-Agresti et al. (2017)

## Live Bacteria as Mucosal DNA Vaccine Delivery Systems: *L. lactis* as a Carrier of DNA Vaccines

Besides the heterologous protein production systems, the use of live bacteria for DNA delivery to eukaryotic cells that will then express the desired proteins has been extensively explored. Such proteins can have prophylactic or therapeutic activities (Guimarães et al., 2009; Tao et al., 2011; Mancha-Agresti et al., 2016; Yagnik et al., 2016).

Live bacteria as vehicles for DNA vaccine delivery have various advantages, such as the capacity to protect the DNA vaccine from degradation by enzymatic action or adverse conditions encountered during transit through the GIT (such as acid pH and high bile and salt concentrations). They also have potential for oral administration, which can stimulate both mucosal and systemic immune responses (Becker et al., 2008; Wang et al., 2016; Yurina, 2018).

**FIGURE 2:**
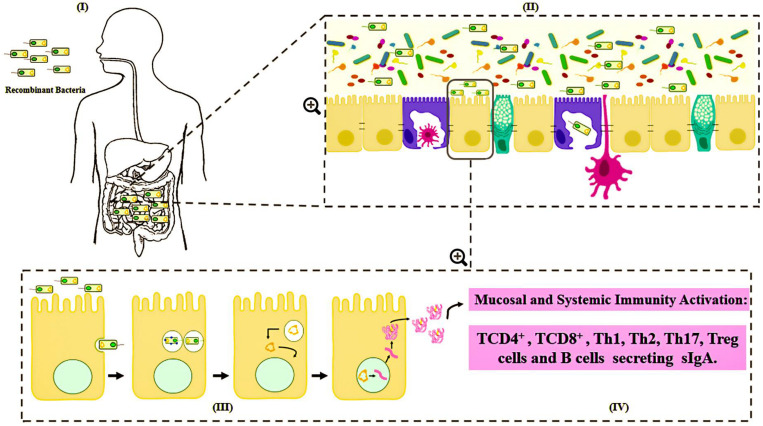
Schematic representation of mechanism proposed for DNA vaccine delivery system into mammalian cells using live bacterial vectors. (I) Oral administration of the recombinant bacteria (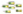); (II) Bacteria in contact with the intestinal surface where they are recognized by the Microfold cells (M cells) (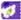), enterocytes (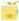), or immune cells [such as dendritic cells (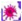)]; (III) The recombinant bacteria is engulfed by the phagolysosome complex and lysed (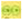). The vaccinal plasmid (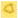) escapes from the vesicle and reaches the nucleus (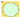) of the host cell. Inside the nucleus transcription of the gene of interest occurs (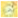); (IV) The protein (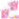) is exposed to the immune system, inducing cellular and humoral immune responses.

Various routes can be used to administrate these bacterial carriers of vaccinal plasmids, such as mucosal routes, including intranasal, oral and genital options (Cortes-Perez et al., 2007; Wells and Mercenier, 2008). However, the mechanisms by which DNA is transferred from live bacterial vectors to eukaryotic cells are not well understood. Among the main hypotheses about the mechanism of action is that after being delivered orally, the bacteria carrying the DNA vaccine enter into contact with the intestinal surface, where they are recognized and phagocytized by either intestinal epithelial cells (IECs), such as the Microfold cells (M cells) and enterocytes, or immune cells, such as the dendritic cells (DC) (Kaiserlian and Etchart, 1999; Weiss and Chakraborty, 2001; Wells J., 2011; Mancha-Agresti et al., 2016; Coelho-Rocha et al., 2018). Pattern recognition receptors (Toll-like and Nod-like receptors) can respond to bacterial components known as microbe-associated molecular patterns (MAMPs), which in turn, serve as natural antigens after host cell invasion (Barbosa and Rescigno, 2010). After entering eukaryotic cells (enterocytes, M cells or dendritic cells), bacteria are usually engulfed by a primary vesicle (phagosome), leading to cell lysis and the release of plasmid DNA. This DNA can then reach the host cytosol and migrate to the nucleus through a net of microtubules and their associated motor proteins in the cytoplasm. In the nucleus, the ORF of interest will be transcribed for subsequent protein synthesis by the host cellular machinery (Weiss and Chakraborty, 2001). The expressed antigen can be presented by either class I MHC to activate the CD8^+^ T cells, or it can be expressed as extracellular protein presented by class II MHC to activate antibody production and the T helper CD4^+^ cell response; thus, inducing cellular and humoral immune specific responses against the encoded antigen (de Azevedo et al., 2015) (Figure 2).

The biodistribution and persistence of a plasmid is dependent on the delivery method and the route of administration. Consequently, the use of a mucosal route and non-pathogenic bacteria as live vectors is promising for vaccine delivery. Also, as these are non-invasive bacteria, they are more acceptable and safe for DNA vaccine delivery.

The relevant regulatory documents for DNA vaccines are from both Europe (EU) and the United States. In July 2019, the WHO (2019) issued a guidance document to assist in the development of DNA vaccines entitled “Guidelines for assuring the quality, safety, and efficacy of DNA vaccines.” This document delineates the manufacturing, preclinical and clinical issues relevant to the development of DNA vaccines and describes potential safety concerns that vaccine developers should address prior to the initiation of clinical studies. These guidelines provide advice to vaccine developers concerning methods for the production and control of DNA plasmids, and they also provide the type of information required for submissions to national control authorities (WHO, 2019).

Based on these guidelines, the DNA vaccine strategy presents various advantages over the more traditional approaches, including more rapid design, ease in improving or adapting plasmid sequences, the possibility of providing multiple vaccines in one injection, and ease in formulating with adjuvants. The DNA can be rapidly isolated and cloned and the production systems are relatively inexpensive and are reproducible in a large-scale production, the bacteria are highly stable and are unable to revert to a pathogenic form (unlike live-attenuated vaccines). They have the ability to induce both humoral and cellular responses and provide immune priming, though they are poor at immune boosting. Another important consideration is the relative ease of large-scale manufacture (Robertson et al., 2007).

Despite these advantages, there is concern about the safety of the use of DNA vaccines. These concerns include the possibilities of long-term persistence, integration into the host genome, thereby increasing the risk of mutagenesis and oncogenesis, vertical and horizontal transmission, induction of auto-immunity by interfering with tolerance to self-antigens or induction of anti-DNA antibodies, altered immune responsiveness to other vaccines and infection, toxicity and immunotoxicity (Saade and Petrovsky, 2012; FDA, 2016.

Various delivery strategies have been studied to increase DNA vaccine efficiency. For DNA vaccines that are naked DNA, chemical delivery systems, such as encapsulation and using of nanoparticles, have successfully increased DNA vaccine delivery; however, they are not able to increase the immune response, as they are inert. Conversely, live non-pathogenic bacterial vectors as DNA carriers overcome some of the disadvantages, as besides functioning as efficient carriers, they also can potentiate an immune response due to their immunogenic features (Saade and Petrovsky, 2012).

The main concern about bacteria as vaccine carriers is safety. Although some attenuated recombinant strains of pathogenic bacteria have been developed, non-pathogenic bacteria such as LAB are considered more suitable as DNA vaccine carriers. The potential risk of using LAB based mucosal vaccines is the escape of genetically manipulated organisms to the environment. Cloning and expression vectors are designed using antibiotic resistance genes as markers for selection. The reengineered bacteria, which produce antigens and antibiotic markers, may allow horizontal transfer of plasmids to other bacteria. Consequently, food grade plasmids and auxotrophic strains, as well as the use of CRISPR/Cas technology, have been designed for avoiding the possibility of horizontal transfer of plasmids that could carry antibiotic resistance genes to environmental and host microflora (Bahey-El-Din et al., 2010; Lin et al., 2012; Berlec et al., 2018).

**FIGURE 3:**
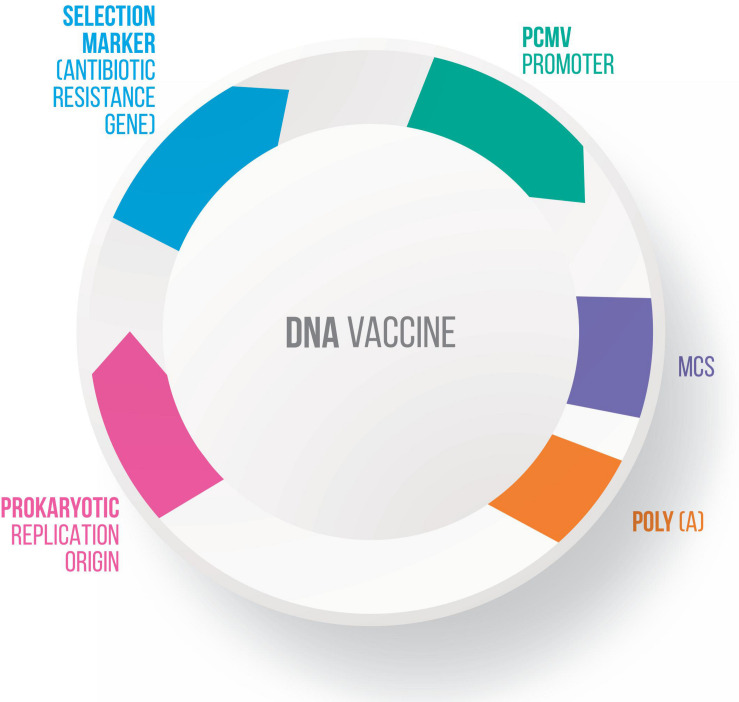
Genetic components of a plasmid DNA vaccine. The essential components in the DNA vaccines includes: a eukaryotic promoter (pCMV), a multiple cloning site (MCS), a polyadenylation site (polyA), a selection marker (antibiotic gene resistance) and a bacterial origin of replication (ori).

Many vectors have been developed for DNA vaccines, using food-grade LAB as live delivery vehicles at the mucosal level (Guimarães et al., 2006, 2009; Tao et al., 2011; Mancha-Agresti et al., 2016; Yagnik et al., 2018). These vectors generally present a series of characteristics in common, including: (i) a eukaryotic promoter (such as pCMV – cytomegalovirus promoter), which allows protein expression by eukaryotic cells; (ii) a multiple cloning site (MCS), where the ORF of the molecule of interest will be inserted; (iii) a prokaryotic region that has a selection marker, usually an antibiotic resistance marker; and (iv) an origin of replication, which ensures that the plasmid replicates only in prokaryotic cells (Kutzler and Weiner, 2008) (Figure 3). Here follow the most recent vectors used for DNA vaccine platforms.

### pLIG Vector

To explore the potential of the *L. lactis* subsp. *cremoris* MG1363 strain as a DNA delivery vehicle, the pLIG vector was constructed. This vector can replicate in both *E. coli* and *L. lactis*, and it contains a eukaryotic expression cassette (Guimarães et al., 2006). The cDNA of bovine β-lactoglobulin antigenic protein (BLG – the most abundant whey protein of cow’s milk and considered a dominant allergen) was cloned in this *L. lactis* strain. This recombinant *L. lactis* MG1363 was used to deliver a eukaryotic expression cassette encoding BLG to mammalian cells. Co-culture of the human epithelial cell line Caco-2 and *L. lactis* (pLIG*:BLG)* confirmed the ability of this microorganism to deliver DNA to mammalian cells, allowing the expression of BLG. When this recombinant strain was administered intragastrically to mice, more than 50% expressed the BLG protein in the epithelial membrane of the small intestine (Chatel et al., 2008).

In order to increase the efficiency of delivery of the eukaryotic expression cassette to epithelial cells, a new strategy was developed. This strategy consisted in the use of a recombinant *L. lactis* strain capable of invading and delivering the vaccine plasmid to epithelial cells (Guimarães et al., 2005). It involved cloning the Fibronectin-Binding Protein A (FnBPA) ORF from *S. aureus* in a prokaryotic plasmid (pOri), converting the *L. lactis* strain into an invasive strain, named *L. lactis* FnBPA*^+^* (Que et al., 2001).

### pValac-Vaccination Using Lactic Acid Bacteria

The pValac plasmid, with a molecular weight of 3742 bp, developed for antigen delivery in lactococci, was constructed by the fusion of a eukaryotic region, so that a molecule of interest can be cloned under the control of the pCMV eukaryotic promoter to be expressed by the host cell, with a prokaryotic region, allowing rolling circle origin replication. Subsequently, the recombinant bacteria are selected by chloramphenicol antibiotic resistance (Guimarães et al., 2009).

Engineered *L. lactis* FnBPA^+^ was used as a vehicle capable of delivering pValac:*IL-10* (the ORF coding *Mus musculus* IL-10) directly to eukaryotic cells. TNBS (del Carmen et al., 2013) or DSS (Zurita-Turk et al., 2014) were used to induce IBD in mice; the animals that received *L. lactis* FnBPA^+^(pValac:*IL-10*) showed lower damage scores in their large intestines (at both macroscopic and microscopic levels), reduced microbial translocation to the liver, and increased anti-inflammatory/pro-inflammatory cytokine ratios compared to mice that received *L. lactis* FNBPA^+^ without the pValac:*IL-10* plasmid.

In order to develop a more effective alternative for Crohn’s disease therapy, the recombinant strain *L. lactis* FnBPA^+^(pValac:*IL-4*) was orally administrated. It was able to reduce the levels of pro-inflammatory cytokines (IL-12, IL-6) and increase the levels of IL-10 and IL-4 secreting regulatory cells (Souza et al., 2016). Similarly, mucosal delivery of *L. lactis* carrying a pValac anti-TNFα scFv expression plasmid in a DSS-induced colitis mouse model significantly improved histological scores and the disease activity index compared to untreated animals (Chiabai et al., 2019).

In addition to treatment/prophylactic approaches related to inflammatory diseases, strategies for vaccination were also tested. Both *L. lactis* FnBPA^+^ (pValac:*ESAT-6*) (Pereira et al., 2015) and *L. lactis* FnBPA^+^ (pValac:*Ag85A*) (Mancha-Agresti et al., 2017) were orally and intranasally administrated, respectively, to mice using an experimental vaccination protocol against tuberculosis (TB). The invasive strain encoding the Esat-6 antigen was able to significantly increase the IFN-γ levels in spleen cells, and also the sIgA in colon tissues. In order to increase the response of *L. lactis* FnBPA^+^ (pValac:*ESAT-6*), the primer-booster strategy was tested. Animals vaccinated with BCG vaccine were boosted with this recombinant strain. Significant increases in splenic pro-inflammatory cytokines (IL-17, INF-γ, IL-6, and TNF-α) were observed in primer-boosted animals (Pereira et al., 2017). The intranasal delivery approach with recombinant *L. lactis* encoding the Ag85A antigen resulted in a significant increase in pro-inflammatory cytokines (IFN-γ, TNF-α, and IL-6) in the stimulated spleen cell supernatants, demonstrating a systemic T helper 1 (Th1) cell response. Antibody production (IgG and sIgA anti-Ag85A) was also significantly increased in bronchoalveolar lavage, as well as in the serum of treated mice (Mancha-Agresti et al., 2017). This demonstrates the effectiveness of this novel DNA delivery therapeutic strategy, with potential for vaccination applications and also for prophylactic treatment.

### pExu – Extra Chromosomal Unit

New DNA vaccine vectors using LAB as delivery vehicles are under development. A new broad range vector, pExu (Extra Chromosomal Unit- 6854 Kb), can be used for DNA vaccine delivery in both *L. lactis* and *Lactobacillus* strains. This plasmid provides a theta-type replication origin and is more stable than previous options. It includes the *erm*AM gene, conferring erythromycin resistance as a selection marker. Its functionality was evaluated by cloning the *egfp* ORF, the expression of which was observed in eukaryotic cells after plasmid transfection. It was also expressed in the small intestine of BALB/c mice after oral administration (Mancha-Agresti et al., 2016). This shuttle vector was successfully inserted into various bacterial species (*E. coli* Top10, *L. lactis* MG1363, *Lactobacillus delbrueckii* CNRZ327 and *L. delbrueckii* CIDCA 133 strains) and stably replicated in all of them.

Applicability of pExu was demonstrated by Coelho-Rocha et al. (2018); they used microencapsulated *L. lactis* strain MG1363 carrying the pExu vector encoding the Red Fluorescent Protein *mCherry* reporter ORF (*L. lactis* pExu:*mCherry*) and investigated the response throughout the GIT at various times post-administration. mCherry expression was observed all parts of the intestine, demonstrating that the pExu vector delivered by *L. lactis* was able to reach the cell nuclei (Coelho-Rocha et al., 2018).

### pPERDBY Vector

The pPERDBY vector (4.9 kb) includes replication origins for both *E. coli* and *L. lactis*, with chloramphenicol as a resistance marker. This vector also includes a multiple cloning site fused with the *egfp* gene (Yagnik et al., 2016); consequently, expression of the cloned gene can be monitored without any additional marker. pPERDBY functionality was evaluated by transfection into mammalian cell lines (CHO-K1 and Caco-2), and co-culture with *L. lactis* NZ900 harboring pPERDBY (Yagnik et al., 2018). Expression of the green fluorescent protein in eukaryotic cells demonstrated its functionality. Tests with BALB/c mice showed that this recombinant bacteria is able to deliver pPERDBY and elicit both systemic and mucosal immune responses (IgG, sIgA, IL-4) against the EGFP antigen, demonstrating its potential as an oral mucosal vaccine carrier (Yagnik et al., 2018).

DNA vaccines are a promising option for vaccination and have already been applied in veterinary medicine. Some characteristics of this vaccination platform are particularly relevant for animal vaccination, including their being relatively inexpensive, since DNA plasmids can be replicated in large amounts by bacteria. They are also stable at room temperature, facilitating transport without requiring any cooling method. Various genes can be delivered simultaneously, and they can elicit both cellular and humoral immune responses (Dhama et al., 2008; Redding and Weiner, 2009; Myhr, 2017). Consequently, DNA vaccines could be very advantageous for preventing or treating animal diseases, especially when the current options are unaffordable or unfeasible (Table 1).

### CRISPR – Clustered Regularly Interspaced Short Palindromic Repeats

In recent years, various genetic tools have been developed and adapted to study and engineer LAB, including controlled gene expression systems, food-grade selection markers, advanced mutagenesis tools, and DNA vaccine vector delivery systems (van der Els et al., 2018; Plavec and Berlec, 2019). Among these genetic tools, the CRISPR-Cas (Clustered Regularly Interspaced Short Palindromic Repeats – CRISPR associated proteins) system has been used for LAB engineering and recombination. This system originates from an efficient phage defense mechanism that is widely disseminated in bacteria and archaea (Millen et al., 2012; Terns, 2018).

Using this technology, Berlec et al. (2018) constructed various plasmids with the intent to induce dual protein expression in *L. lactis*, confirmed by the expression of two proteins, the infrared fluorescent protein (IRFP) and DARPin I07, which has high affinity for human IgG. Modifications were made to construct an inducible single-plasmid CRISPR-Cas9 and CRISPRi (CRISPR interference system) capable of producing an sgRNA and Cas9 or dCas9 (Cas9 Endonuclease Dead, a mutant form of Cas9 in which the endonuclease activity has been removed) concomitantly (Berlec et al., 2018). Improvements in the methods for genetic modifications of LAB enable new approaches for both food and health product production.

Other CRISPR-Cas applications in LAB include genomic island targeting and phage editing in *Streptococcus thermophilus* (Martel and Moineau, 2014; Selle et al., 2015), Cas9 nickase-variant-driven chromosomal insertions in *L. casei* (Song X. et al., 2017), Cas9-assisted recombineering in *L. reuteri* (Oh and van Pijkeren, 2014), and plasmid curing in *L. citreum* (Jang et al., 2017).

In *L. reuteri*, the CRISPR-Cas system was combined with ssDNA (single-strand DNA) recombineering to enhance its performance by promoting cotransformation of a CRISPR-target plasmid and a recombineering oligonucleotide in a single-step, increasing the production of recombinants when ssDNA recombineering efficiency is optimized. In this process, Cas9 was directed to the wild-type sequence of the genome, killing the bacterial cells that were not modified by recombineering, thereby avoiding the need for mutation screening (Oh and van Pijkeren, 2014).

Two methods, both employing Cas9 to cleave an unmodified genomic DNA, were compared in various *L. plantarum* strains. The first method utilized a plasmid-encoded homology template, and the second used oligonucleotide-based recombineering. The relative efficacy of the two methods differed among *L. plantarum* strains, highlighting the importance of considering and testing various approaches for genome engineering (Leenay et al., 2019).

Zhou et al. (2019), in an attempt to increase protein production without inserting exogenous genes, used CRISPR/Cas9 to engineer *L. plantarum* strain WCFS1 to produce N-acetylglucosamine (GlcNAc). This was achieved by truncating the gene *nag*B, which eliminates the reversion of fructose-6-phosphate (F6P) back to glucosamine-6P. This recombinant *L. plantarum* WCFS1 was able to produce 797.3 mg/L *N*-acetylglucosamine (GlcNAc) by inducing the GlcNAc pathway. This genetic edition facilitated industrial production of this protein and demonstrated the potential of this genetic engineering technique for application in other organisms (Zhou et al., 2019).

A more recent work (Xiong et al., 2020) used CRISPR/dCas9 with the objective to repress genes without disrupting their nucleotide sequences. Two plasmids (pKLH116 – containing the Cas9 and the P_nisin_ promoter, and pSGRNAs containing the sgRNA and the P_44_ promoter) were constructed and inserted into *L. lactis* NZ9000. To test their efficiency, sgRNA was constructed targeting six different regions of three genes: *upp*, which metabolizes 5-fluorouracil, *sod*, which is involved in oxidative stress control, and *LLNZ_07335*, which encodes a putative penicillin acylase. This system was able to repress the *upp* gene by 70 to 98%, the *sod* gene by 48 to 80%, and the *LLNZ_07335* gene by 9.3 to 67.2%. Xiong et al. (2020) also tested the capability of the system to repress more than one gene at once. They used a plasmid with sgRNA for the *upp* gene and the *LLNZ_07335* gene; this combination resulted in a lower rate of repression when compared to the repression by one gene alone. They suggested that this reduction in repression is due to competition for the Cas9 active site. They concluded that this repression system has the potential to evaluate gene function; though it also could be used for metabolic engineering and synthetic biology involving other LAB species and strains (Xiong et al., 2020).

The adaptation of CRISPR for modifying LAB is a promising option for engineering recombinant organisms for heterologous protein production and as DNA vaccine vehicles. The wide range of possibilities and the simplicity of the CRISPR-Cas system should facilitate the development of new and more efficient plasmids and expression systems.

## Targeting of *Lactococcus lactis* to Specific Host Cells

In order for LAB to play their role as DNA delivery vectors, they must reach the target site in sufficient quantities. However, the viability of these bacteria drops significantly when they are exposed to adverse conditions, such as the extremely acidic environment of the stomach and bile salts in the small intestine. Facilitating interactions between LAB and the host mucosa/epithelia could help prolong the retention of bacteria on mucosal surfaces and thereby enhance the therapeutic effects of recombinant LAB. Increased adhesion can be achieved by expressing adhesion factors or by fusing the bacteria to the therapeutic proteins (Michon et al., 2016).

Along this line an invasive *L. lactis* strain expressing the *Listeria monocytogenes* internalin A (InlA) gene was developed. InlA encodes an 84 kDa protein that is anchored to the bacterial cell wall, mediating the entry of this microorganism into mammalian epithelial cells by binding to E-cadherins in these cells (Gaillard et al., 1991; Lebrun et al., 1996). The InlA anchored to the cell envelope of *L. lactis* promotes its internalization and, consequently, plasmid delivery. The effectiveness of internalization of this bacteria, promoting eukaryotic expression of the GFP reporter, was demonstrated *in vitro* in a Caco-2 cell line and *in vivo* through oral administration in guinea pigs (Guimarães et al., 2005). However, while attractive, this approach has a major limitation in mouse models, since InlA does not interact with the murine E-cadherin receptor. Thus, the strategy of using recombinant *L. lactis* InlA as a vehicle for DNA delivery can only be tested in guinea pigs expressing human E-cadherin or in transgenic mice (Lecuit et al., 2001).

An alternative that overcomes this disadvantage of not interacting with the cadherin receptor was developed; recombinant *L. lactis* strain expressing *S. aureus* FnBPA (Que et al., 2001) was tested for DNA delivery to mammalian cells (Innocentin et al., 2009). FnBPA mediates bacterial adhesion to host tissue and entry into non-phagocytic cells. As a consequence, recombinant strain *L. lactis* FnBPA^+^ was able to invade cells, in an *in vitro* test, at levels comparable to *S. aureus* (Sinha et al., 2000). In addition, the invasiveness and the delivery capability of *L. lactis* expressing FnBPA protein were comparable to *L. lactis* expressing InlA when tested in the Caco-2 human epithelial cell line, and definitely superior to the non-invasive *L. lactis* strain (Innocentin et al., 2009).

This superior *in vitro* delivery capability was confirmed in tests with the pValac:*blg* plasmid. When Caco-2 cells were co-incubated with *L. lactis* FnBPA^+^ (pValac:*blg*), they produced up to 30 times more BLG than Caco-2 cells co-incubated with the non-invasive strain (Pontes et al., 2012). In addition, mice that consumed *L. lactis* FnBPA^+^ (pValac:*gfp*) expressed GFP in small intestine (Pontes et al., 2012) and large intestine cells (del Carmen et al., 2013).

FnBPA has also been shown to be an adjuvant for co-delivered antigens; however, the underlying mechanisms have not been elucidated. In another study, the invasive *L. plantarum* with surface displayed FnBPA were able to modulate the host immune response by stimulating differentiation of dendritic and T helper cells, which could be responsible for the adjuvant effects of FnBPA (Liu et al., 2019).

The disadvantages of the approach expressing this adhesin heterologously needs to be considered. Staphylococcal FnBPA allows the colonization of heart valves by otherwise non-pathogenic *L. lactis*; it also promotes dissemination into the spleen (Que et al., 2005; Piroth et al., 2008). The role of FnBPA in virulence is supported by *in vitro* studies that show that *L. lactis* expressing FnBPA is able to activate endothelial cells, inducing inflammatory and pro-coagulant responses (Heying et al., 2007, 2009).

The finding that *L. lactis* IL1403 can produce and secrete recombinant murine IL-6 when fused with M cell-targeting moieties demonstrated that recombinant bacteria can serve as efficient adjuvants for oral vaccination (Li et al., 2015). Similarly, the M cell-targeting moiety and the viral capsid protein 2 antigen of infectious bursal disease virus co-expressed by *L. lactis* NZ3900 elicited a high degree of immunoreactivity in chickens (Liu et al., 2018). Thus, apart from increasing intestinal retention, specific immune cell types can also be targeted by this delivery system.

As an alternative, encapsulation methods have been tested to protect bacteria against unfavorable conditions faced in their passage through the GIT (Sultana et al., 2000; Riaz and Masud, 2013; Coelho-Rocha et al., 2018). According to Lakkis (2007), encapsulation technology is defined as a process that traps one substance inside another, producing particles on a nanometer (nanoencapsulation), micrometer (microencapsulation) or millimeter scale. The material to be used for oral administration purposes must be GRAS and capable of forming a barrier between the internal compound and its surroundings. Sodium alginate is a suitable material for this procedure and is compatible with almost all types of encapsulations (Burgain et al., 2011).

Research has shown that LAB encapsulation increases viability in dairy products without changing product color, acidity or taste (Rosa et al., 2013). This methodology was also applied to optimize the transfer of vaccine plasmids into intestinal cells, as described by Coelho-Rocha et al. (2018). They constructed a recombinant *L. lactis* (pExu:*mCherry*) and encapsulated it with 1% sodium alginate. Bacterial encapsulation increases bacterial viability, allowing more bacteria to reach further regions of the intestine, being an effective method for improving plasmidial DNA delivery (Coelho-Rocha et al., 2018).

## Safety Aspects

Recombinant *L. lactis* are genetically modified organisms (GMO) and are therefore treated with caution by regulatory agencies and the public, despite the known safety of wild-type *L. lactis.* Proper biosafety precautions should be taken in accordance with established biosafety standards and regulations. All materials that have been used must be autoclaved (121°C, 15 min) and disposed of in accordance with current country regulations, such as directives from the Food and Drug Administration (FDA-USA), European Union Law (EUL – EU) and the Office of the Gene Technology Regulator (Department of Health – Australia). GMOs can be classified into four classes according to potential risk to the environment, including plants, humans, and other animals, with different rules for each risk class.

*Lactococcus lactis* are able to survive the passage through the GIT after oral administration. To prevent the spread of modified bacteria into the environment, recombinant *L. lactis* strains that allow biological containment have been developed. To this end, auxotrophic bacteria have been developed by the elimination of essential genes involved in the production of key metabolites such as amino acids (Ronchel and Ramos, 2001) and nucleosides (Steidler et al., 2003; Bahey-El-Din and Gahan, 2010). Notwithstanding, these metabolites may be available from the environment, produced by other organisms, allowing the survival of engineered bacteria (Lim and Song, 2019).

Another important detail that should be taken into consideration is that many species of the genera *Lactobacillus, Leuconostoc, Pediococcus, Enterococcus*, and *Bifidobacterium* have been isolated from pathological conditions, such as bacterial endocarditis, systemic infections, and meningitis (Liong, 2008). Bacterial translocation from the intestine generally involves immunosuppression and reduction in the intestinal barrier, resulting in the passage of bacteria from the intestine to the blood stream, which can lead to bacteremia, septicemia, and multiple organ failure (Berg, 1992). Most LAB strains involved in such clinical cases are *Enterococcus faecium* and *E. faecalis*, with a few reports involving *L. rhamnosus*, *L. casei, L. paracasei*, and *L. plantarum* (Gasser, 1994). In healthy individuals, bacterial translocation has been found to occur frequently, without any deleterious consequences, at a rate of 5–10% (Sedman et al., 1994). Studies involving healthy individuals have not reported severe diseases such as sepsis caused by probiotic translocation. The explanation for this phenomenon is not fully understood; possibly this is due to the susceptibility of probiotics to attack by macrophages outside the intestine (Duffy, 2000; Veltrop et al., 2000).

Although DNA vaccines are a promising strategy to protect against a wide variety of diseases and have many advantages in comparison with other methodologies, concerns have risen about safety for the host and environmental issues regarding GMO. However, using LAB as vaccine carriers has shown promise because of their well-known properties. LAB have been extensively used in the food industry and consequently have been tested for their immunomodulatory capabilities, obtaining a safe status. Also, LAB can induce cytokine production in both Th1 and Th2 cells, properties useful for vaccine carriers (Hanniffy et al., 2004; Detmer and Glenting, 2006; Lee, 2010).

Due to the gastrointestinal resistance of LAB compared to other types of bacteria, DNA vaccines based on recombinant LAB could potentially result in the dissemination of these bacteria into the environment, especially non-colonizing strains such as *L. lactis*. The use of these GMO raises justifiable concerns about their survival and propagation in the environment, possibly resulting in the spread of antibiotic selection markers or other genetic modifications to other microorganisms. Microorganisms have evolved highly efficient systems for horizontal gene transfer, such as transformation, conjugation, retro-mobilization, and transduction to improve their adaptation to changes in the environments they colonize. A high transfer frequency of both erythromycin and tetracycline-resistance has been observed between LAB and related species (Lee, 2010).

A food-grade expression system has been tested as an alternative to avoid the dispersal of antibiotic markers through GMOs intended for practical application. In this system, instead of the commonly used antibiotic selection marker, a modification is made in an essential bacterial gene, thus providing a presumption of safety status that has potential for food production and vaccine development without the risk of horizontal transfer of antibiotic resistance (Lu et al., 2016). To qualify as a safe product, a detailed description of the DNA plasmid is compulsory and must include information concerning the origin and nucleotide sequence of the gene(s) encoding the protein (or peptide), the selection marker, the identity of the microorganism or organism from which the gene was derived, and the origin of the microorganism that will be used (Klug et al., 2012).

There are other safety aspects that need to be observed. Biodistribution and persistence are evaluated by looking for the DNA vaccine in different organs of the host and investigating the persistence of the plasmid DNA in the body; these factors concern the risk for germ line transmission (Lee et al., 2018). Unintended immune responses of DNA vaccination could include autoimmune reactions and intrinsic immunostimulatory activity, inducing the production of proinflammatory cytokines (Lee et al., 2018). Vaccine developers need to evaluate the potential to integrate into the host’s genome, resulting in disruption of host gene expression (Faurez et al., 2010; Myhr, 2017). DNA vaccines may cause indel mutations, the risks of which depend on the mechanism of delivery (Lee et al., 2018). Though bacterial gene delivery systems are safer than those that use viral vectors, the risks of insertional mutagenesis are not zero; however, the risks are believed to be lower than those of everyday random mutations (Lee et al., 2018). Native LAB are considered demanding microorganisms that require considerable nutritional supplementation for their growth, as they are adapted to complex organic substrates, which can be costly to provide (Morishita et al., 1981; Hebert et al., 2000; Hayek and Ibrahim, 2013). The ability of the microorganisms to support extreme conditions, such as extreme temperatures and pH, have a high metabolic cost (Fadda et al., 2010). Recombinant strains in culture initially multiply slower than wild strains; consequently, they take longer to get to the stationary phase. Another important consideration for these strains is the presence of phages, which could represent a risk. In a recent bioinformatics study of 30 complete genomes 84 complete, 51 incomplete, and 31 uncertain prophage regions were found. In this study, Kelleher et al. (2018) emphasize the low risk of prophage induction as these regions are stable residents of the bacterial chromosome.

## Final Considerations

Lactic acid bacteria, such as *L. lactis*, are the most widely used organisms in the food industry. In recent decades, they have also become biotechnological tools. The large variety of plasmids and expression systems designed for these organisms has made them promising for heterologous protein production and as DNA vaccine delivery vectors, with advantages over other candidate organisms, such as *E. coli*, due to their safe status and because they do not produce toxins. New technologies, such as CRISPR, have taken LAB bioengineering to a higher level, providing new and more efficient plasmids and expression systems, as well as modified bacteria. There are still some concerns regarding the safety of the release of these modified organisms in nature and their use and long-term effects in humans, though many researchers have been working to overcome these issues. With the rapid and continuous evolution of the relevant technology, LAB have the potential to become the organisms of choice for protein expression and DNA delivery, overcoming current barriers and providing a safer and easier to apply alternative.

## Author Contributions

LT, LJ, TS, FB, VB, and NC-R wrote the original draft of the manuscript. VA, MD, and PM-A wrote and reviewed the manuscript, obtained the funding, and supervised the project. All authors contributed to the article and approved the submitted version.

## Conflict of Interest

The authors declare that the research was conducted in the absence of any commercial or financial relationships that could be construed as a potential conflict of interest.
